# EMPOWER!: Brain Health Education to Promote Early Detection of Alzheimer’s and Cognitive Screening

**DOI:** 10.1093/geroni/igaf122.1202

**Published:** 2025-12-31

**Authors:** Veronica Derricks, Sarah VanHeiden, Jane Musema, Miriam Jocelyn Rodriguez, Patricia Garcia, Sophia Wang, Christopher Carey

**Affiliations:** University of Colorado Boulder, Boulder, Colorado, United States; Indiana Alzheimer’s Disease Research Center, Indianapolis, Indiana, United States; Indiana Alzheimer’s Disease research center, Indianapolis, Indiana, United States; Indiana University Bloomington, Bloomington, Indiana, United States; Indiana University School of Medicine, Indianapolis, Indiana, United States; Indiana Alzheimer’s Disease research center, Indianapolis, Indiana, United States; Indiana University School of Medicine, Indianapolis, Indiana, United States

## Abstract

**Introduction:**

Black older adults are twice as likely to develop Alzheimer’s disease and related dementias (ADRD). They are also more likely to have delayed or missed diagnoses of ADRD, because of lower cognitive screening rates during the earliest stages of the disease. To address these gaps, we developed the EMPOWER! program to provide education about the importance of early detection of ADRD for those communities at higher risk of this disease.

**Methods:**

Educational materials about brain health and the importance of cognitive screening were co-developed with community stakeholders. Qualitative interviews were conducted with Black older adults who had cognitive concerns and <16 years of education, their close contacts, and community center staff working closely with communities at risk of ADRD (N = 15). Thematic analysis identified key themes related to barriers, facilitators, and recommendations for how to improve relevance and accessibility to information about brain health, AD, and cognitive screening.

**Results:**

Study results highlighted the critical factors influencing cognitive health awareness and research participation among Black older adults. Findings emphasize the importance of trust, social support, culturally relevant communication, and targeted outreach in encouraging engagement with cognitive screening and brain health initiatives.

**Discussion:**

Our preliminary data identified factors which may increase acceptability of culturally tailored materials to engage communities most at risk of ADRD to learn more about brain health and the importance of early detection. Future efforts will further assess content and attitudes towards cognitive screening and adapting EMPOWER! for use in other communities at risk of ADRD.

